# Artemisinin Protects Porcine Mammary Epithelial Cells against Lipopolysaccharide-Induced Inflammatory Injury by Regulating the NF-κB and MAPK Signaling Pathways

**DOI:** 10.3390/ani11061528

**Published:** 2021-05-24

**Authors:** Wenfei Zhang, Liang Xiong, Jiaming Chen, Zhezhe Tian, Jiaxin Liu, Fang Chen, Man Ren, Wutai Guan, Shihai Zhang

**Affiliations:** 1 Guangdong Provincial Key Laboratory of Animal Nutrition Control, College of Animal Science, South China Agricultural University, Guangzhou 510642, China; zhangwenfei@stu.scau.edu.cn (W.Z.); jhyxl15170466109@163.com (L.X.); 20193139005@stu.scau.edu.cn (J.C.); tzz197211@stu.scau.edu.cn (Z.T.); 20193139038@stu.scau.edu.cn (J.L.); chenfang1111@scau.edu.cn (F.C.); 2 National Engineering Research Center for Breeding Swine Industry, College of Animal Science, South China Agricultural University, Guangzhou 510642, China; 3 Anhui Provincial Key Laboratory of Animal Nutritional Regulation and Health; College of Animal Science, Anhui Science and Technology University, Fengyang 233100, China; renman@yeah.net

**Keywords:** porcine mammary epithelial cells, lipopolysaccharide, anti-inflammation, artemisinin

## Abstract

**Simple Summary:**

Sow mastitis is a serious breast disease that can cause severe inflammation, agalaxia and even lead to death of piglets. Porcine mammary epithelial cells (pMECs) are the main cell types that affect sow milk secretion, therefore, when swine mastitis occurs, the inflammatory response of pMECs directly affects the mammary gland health and sow’s lactation ability. Promoting the health of mammary gland epithelial cells is an important method for treating mastitis. Thus, in the current study, we investigated the effects of artemisinin on the inflammatory response of pMECs induced by lipopolysaccharide (LPS), and proposed a potential anti-inflammatory mechanism. We confirmed that artemisinin can reduce the inflammatory damage of pMECs induced by LPS by inhibiting MAPK and NF-κB signaling pathways. Pretreatment of pMECs with artemisinin showed enhanced anti-inflammatory activity against LPS-induced inflammation. Artemisinin could be a useful, safe and natural anti-inflammatory feed additive to prevent sow mastitis.

**Abstract:**

Artemisinin performs a variety of biological functions, such as anti-cancer, anti-inflammatory, anti-viral, and anti-oxidant effects. However, the effects of artemisinin on sow mastitis have not been studied. The results of the current study showed that mRNA expression abundance and content of the inflammatory factors interleukin-1β (IL-1β), tumor necrosis factor α (TNF-α), and interleukin-6 (IL-6) were significantly increased when using 50 μg/mL LPS to stimulate pMECs for 24 h (*p* < 0.05). Pretreatment with 20 μM artemisinin weakened LPS-induced inflammatory damage in pMECs and decreased mRNA expression abundance and the content of inflammatory factors (IL-1β, IL-6, and TNF-α) in pMECs (*p* < 0.05). Mechanistically, artemisinin inhibited LPS-induced activation of the mitogen-activated protein kinase (MAPK) and nuclear factor-κB (NF-κB) signaling pathways. In summary, the pretreatment of pMECs with artemisinin showed enhanced anti-inflammatory activity against LPS-induced inflammation.

## 1. Introduction

Mammalian mastitis is one of the common diseases worldwide, which leads to increased veterinary expenses, and significant economic losses to the breeding industry every year [1,2]. The clinical signs of mammalian mastitis are agalaxia (decreases in milk production and quality) and dolor, which are accompanied with increased inflammatory mediators in milk. During mammalian mastitis, piglets can develop hypoglycemia and hypothermia, which might finally lead to death [3,4].

Breast tissue contains large numbers of mammary epithelial cells for milk synthesis [5,6], which can also react with pathogens. This response is important for determining the outcome of mammary gland infection [7]. A large number of studies have shown that the inflammatory response of mammary epithelial cells induced by lipopolysaccharide (LPS) can not only reduce milk production and the levels of fat and protein in milk, but also destroy the milk–blood barrier [8,9,10]. Therefore, it is important to control and reduce the inflammation of mammary epithelial cells during mastitis. Although the use of antibiotics is still an effective way to treat animal mastitis, the use of antibiotics is severely restricted due to the emergence of more and more serious bacterial resistance and food safety [11]. It is important to seek novel alternatives to antibiotics for the effective and safe treatments of mastitis in veterinary research. Chinese herbal medicine with anti-inflammatory activity is a potentially effective option for the treatment of mastitis [12].

Artemisinin (chemical formul, C_15_H_22_O_5_; molecular mass, 282.34 g/moL) is a sesquiterpene lactone compound with a peroxy bridge structure [13]. This specific structure is an important reason for artemisinin and its derivatives being believed to have antimalarial and antibacterial activities [14]. This chemical is isolated from the Chinese plant *Artemisia annua* L. and is effective for the treatment of severe and multidrug-resistant malaria [15,16,17]. Previous studies have shown that artemisinin has a variety of biological functions, such as anti-bacterial [18], anti-inflammatory [19], anti-viral [20], antioxidant [21,22,23], and immunomodulatory activities [24,25]. Although many studies have described the anti-inflammatory functions of artemisinin, the effects of artemisinin on LPS-induced inflammatory damage in porcine mammary epithelial cells (pMECs) remain to be clarified. We hypothesized that artemisinin might have a protective effect on the LPS-induced inflammatory response of pMECs.

## 2. Materials and Methods

### 2.1. PMECs Isolation, Cell Culture, and Treatments

pMECs were isolated from the mammary gland of a lactating sow as previously described [26] and keep refrigerated in liquid nitrogen. pMECs were cultured in Dulbecco’s modified Eagle’s medium/F12 nutrient mixture (DMEM/F12) supplemented with 5 μg/mL hydrocortisone, 10% fetal bovine serum (FBS), 10 ng/mL insulin-like growth factors-1 (IGF-1), 5 μg/mL insulin–transferrin–selenium, 10 ng/mL epidermal growth factor, and 100 U/mL antibiotics (streptomycin and penicillin) at 37 °C in a humidified atmosphere with 5% CO_2_. Medium was changed every 48 h, and cells were subcultured by trypsin digestions at a subcultivation ratio of 1:2.

LPS (lipopolysaccharide, Escherichia coli serotype O55:B5, Sigma, Waltham, MA, USA) was diluted in DMEM/F12. Artemisinin (purity > 99%, The National Institutes for Food and Drug Control, Beijing, China) was dissolved in DMSO (Sigma, Waltham, MA, USA) and diluted in DMEM/F12.

### 2.2. Cell Viability Assay and Flow Cytometric Analysis

To test the cell viability, equal amounts of pMECs were seeded in 96-well plates at a density of 2 × 10^4^ cells/mL and cultured in DMEM/F12 containing 10% FBS. After cultured for 48 h, cells are washed three times with PBS, and then treated with different concentrations of artemisinin or LPS for different times. The cell counting kit - 8 (Nanjing Jiancheng Bioengineering Institute, Nanjing, China) was used to analyze cell viability according to the instruction. An automatic microplate reader (Bio-Rad Laboratories Inc., Foster City, CA) was used to measure the absorbance of all wells at a wavelength of 450 nm. Cell viability is the quantification of No. live cells and is expressed as a percentage of the control.

To test cell apoptosis, pMECs were pretreated with 0 or 20 μM of artemisinin for 12 h before being stimulated with 50 μg/mL LPS for 24 h. After treatment, all cells were trypsinized, washed 2~3 times with PBS, stained with Annexin V-FITC/PI (Invitrogen Inc., Carlsbad, CA, USA), and analyzed by flow cytometry, according to the instructions.

### 2.3. Real-Time PCR

According to the experimental requirements, pMECs were treated with LPS or artemisinin, and then the mRNA expression of inflammatory cytokines and apoptosis-related genes were measured by real-time PCR as described previously [27]. Real-time PCR was performed using the ABI StepOnePlus™ real-time PCR system (Applied Biosystems, Grand Island, NE, USA). The amplification of real-time PCR was performed with a reaction volume of 20.0 μL. Samples were analyzed in duplicate.

Real-time PCR reaction protocol: heating at 94 °C for 5 minutes for initial enzyme activation, then performing 40 cycles at 94 °C for 30 s, and then annealing at 60 °C for 30 s, last extension at 72 °C for 20 s. In this study, β-actin was stability expressed among different treatments, which was selected as a reference gene by geNorm 3.5 (http://medgen.ugent.be/~jvdesomp/genorm. accessed on 10 December 2009) in pMEC [28]. The expression level of each gene was calculated according to the 2^−ΔΔCt^ method [29]. Table 1 shows the primer sequences of target genes.

### 2.4. Measurement of Inflammatory Factor Levels

Cell culture and treatments were performed as described previously [30]. The culture medium in the 6-well plate was aspirated and discarded, the cells were washed with precooled PBS 3 times, and 200 μL of RIPA lysis buffer was added to every well, which was pipetted several times to fully lyse the cells. The lysate was transferred to a 2.0 mL centrifuge tube and centrifuged at 10,000 rpm for 10 min, and the cell supernatant was used to analyze the content of inflammatory factors. Cell supernatants were subsequently used for inflammatory factor analysis with a pig ELISA kit (TNF-α, #CSB-E16980p; IL-1β, #CSB-E06782p; IL-6, #CSB-E06786p), Cusabio Biotech Company, Wuhan, China) according to the manufacturer’s instructions.

### 2.5. Western Blot Analysis

Western blotting was performed as previously described [31]. Proteins were extracted from pMECs using the radio immunoprecipitation assay (RIPA) lysis buffer (#P0013B, Beyotime, Shanghai, China) and quantified using a BCA protein assay kit (#P0009, Beyotime, Shanghai, China). Proteins (10–20 μg/sample) were separated by SDS-PAGE (Invitrogen Inc.), transferred to nitrocellulose membranes (Millipore, Bedford, MA, USA.), and then hybridized with specific antibodies.

Primary antibodies for TLR-4 (1:500, ab13556), p65 (1:1000, ab16502), phospho-p65 (1:1000, ab183559)), β-actin (1:1000, ab5694), p38 (1:1000, ab182453), phospho-p38 (1:1000, ab207483), ERK (1:1000, ab32537), phospho-ERK (1:1000, ab207470), JNK (1:1000, ab126424), and phospho-JNK (1:1000, ab279842) were from the Abcam Company Ltd. (Cambridge, MA, USA). Primary antibodies (dilution, cat. no. follow in parentheses) for IκBα (1:1000, #9242), and phospho-IκBα (1:1000, #2859) were from the Cell Signaling Technology (Woburn, MA, USA).

### 2.6. Statistical Analysis

All data are derived from at least 3 independent experiments performed in triplicate, and the results are expressed as mean ± SEM. One-way ANOVA was used for statistical analysis, and then Duncan’s test was used for multiple comparisons. *p* < 0.05 was used as the criterion for judging the significance of the difference, and *p* < 0.01 was the criterion for judging the extremely significant difference.

## 3. Results

### 3.1. Inflammatory Injury in LPS-Induced PMECs

**Figure 1:**
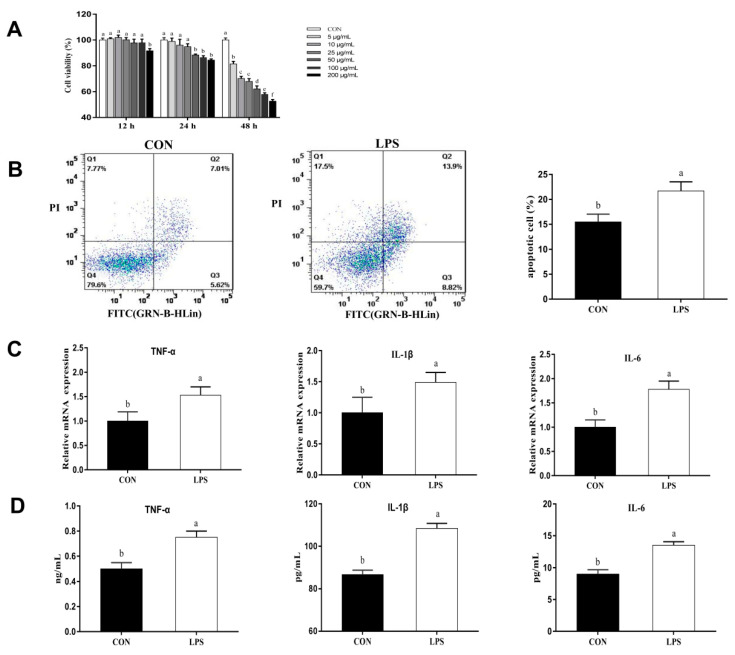
Inflammatory injury in LPS-induced porcine mammary epithelial cells (pMECs). (**A**) Cell viability was measured by CCK-8 following treatment with different concentrations (5, 10, 25, 50, 100, and 200 μg/mL) of lipopolysaccharide (LPS) for 12, 24, and 48 h. The data are the means ± SEM (*n* = 6 to 12). Different superscript letters (a,b,c) indicate that there are significant differences between the groups (*p* < 0.05). CON = control cells without any treatment. (**B**) After pMECs were treated with 50 μg/mL LPS for 24 h, the percentage of cells undergoing apoptosis was determined by flow cytometry and annexin V/propidium iodide (PI) staining. (**C**) Effects on the mRNA expression of inflammatory factors (IL-1β, TNF-α, and IL-6) after pMECs were treated with 50 μg/mL LPS for 24 h. (**D**) Effects on the levels of inflammatory factors after pMECs were treated with 50 μg/mL LPS for 24 h.

In order to determine the effect of inflammatory damage on LPS-induced pMEC, cell viability was measured by the CCK-8 assay after the pMECs were treated with various concentrations (5, 10, 25, 50, 100, and 200 μg/mL) of LPS for 12, 24, and 48 h. As shown in Figure 1A, cell viability was significantly inhibited (*p* < 0.05) after stimulation with 200 μg/mL LPS for 12 h. Cell viability was also significantly inhibited (*p* < 0.05) after stimulation with 50, 100 or 200 μg/mL LPS for 24 h. In addition, the cell viability was significantly decreased (*p* < 0.05) after stimulation with all concentrations of LPS for 48 h. As shown in Figure 1B, after pMECs were treated with 50 μg/mL LPS for 24 h, cell apoptosis was significantly increased (*p* < 0.05), and mRNA expression abundance and the content of inflammatory factors (IL-1β, TNF-α, and IL-6) were also significantly increased (*p* < 0.05; Figure 1C,D). Thus, after comprehensive consideration of cell viability, apoptosis and inflammatory factors, 50 μg/mL LPS for 24 h was selected to establish inflammatory injury in pMECs for subsequent experiments.

### 3.2. Effects of Artemisinin on the Viability of PMECs

**Figure 2:**
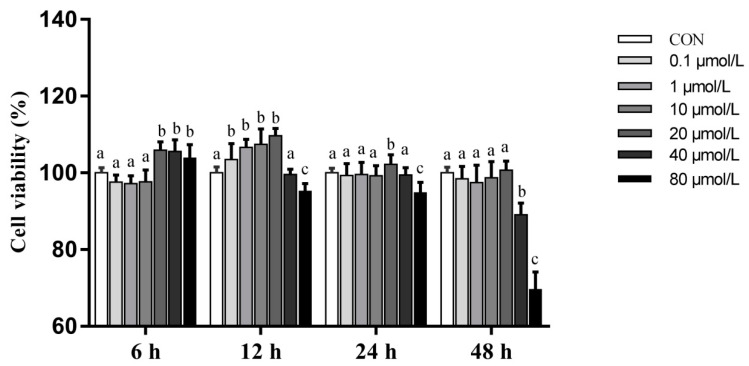
The effects of artemisinin on the viability of pMECs. Cells were treated with different concentrations (0.1, 1, 10, 20, 40, and 80 μM) of artemisinin for 6, 12, 24, and 48 h. Cell viability was determined by the CCK-8 assay. Different superscript letters (a,b,c) indicate that there are significant differences between the groups (*p* < 0.05).

In order to investigate the effect of artemisinin on pMECs viability, cells were treated with various concentrations (0.1, 1, 10, 20, 40, and 80 μM) of artemisinin for 6, 12, 24, and 48 h and analyzed by CCK-8 assay. As shown in Figure 2, low concentrations of artemisinin promoted cell growth and induced cell death in a concentration-dependent manner at higher concentrations. Maximum cell viability significantly increased (*p* < 0.05) when pMECs were treated with 20 μM artemisinin for 12 h. According to these results, 20 μM artemisinin (treated with 12 h) was chosen to examine the protective effects of artemisinin against inflammatory injury in LPS-induced pMECs.

### 3.3. Effects of Artemisinin on Viability and Apoptosis in LPS-Induced PMECs

**Figure 3:**
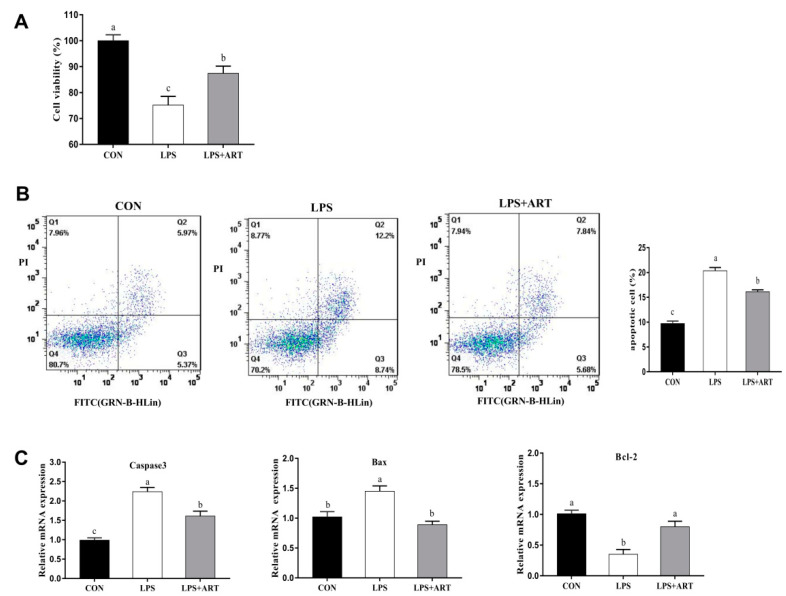
The effects of artemisinin on cell viability (**A**) and apoptosis (**B**,**C**) in LPS-induced pMECs. Cells were pretreated with the indicated concentrations (0 and 20 μM) of artemisinin for 12 h before being stimulated with 50 μg/mL LPS for 24 h. Cell viability was measured by the CCK-8 assay. The percentage of cells undergoing apoptosis was determined by flow cytometry and annexin V/propidium iodide (PI) staining. The mRNA expression levels of apoptotic genes (Caspase-3, Bax, and Bcl-2) were quantified using reverse-transcription PCR. Different superscript letters (a,b,c) indicate that there are significant differences between the groups (*p* < 0.05). CON = control cells without any treatment; LPS = cells treated with only 50 μg/mL LPS; LPS + ART = LPS (50 μg/mL) + artemisinin (20 μM).

In order to determine the protective effect of artemisinin on inflammatory injury in LPS-induced pMECs, cell viability and apoptosis were measured after treatment with artemisinin or LPS. As shown in Figure 3, the LPS-induced decrease in cell viability was significantly inhibited (*p* < 0.05) by 20 μM artemisinin (Figure 3A), and LPS-induced pMECs apoptosis was significantly reduced (*p* < 0.05) by artemisinin (Figure 3B,C).

### 3.4. Effects of Artemisinin on LPS-Induced Inflammatory Factors

**Figure 4:**
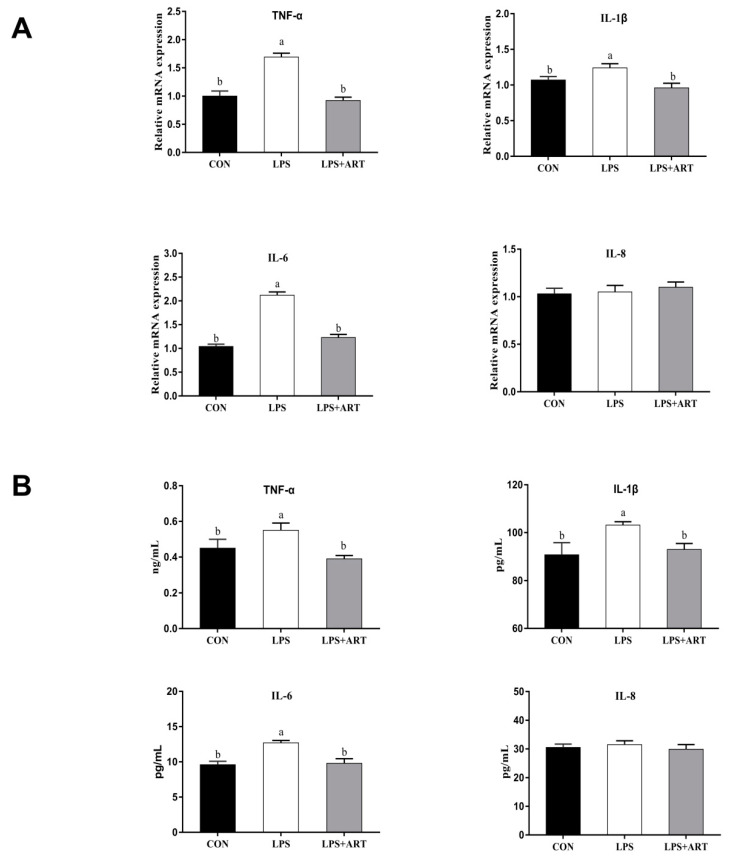
The effects of artemisinin on LPS-induced inflammatory factor (IL-1β, TNF-α, IL-6, and IL-8) gene expression (**A**) and protein expression (**B**). Cells were pretreated with the indicated concentrations (0 and 20 μM) of artemisinin for 12 h before being stimulated with 50 μg/mL LPS for 24 h. The mRNA expression of inflammatory factors (IL-1β, TNF-α, IL-6, and IL-8) was quantified using reverse-transcription PCR, and the protein levels were measured by ELISA. Different superscript letters (a,b,c) indicate that there are significant differences between the groups (*p* < 0.05). CON = control cells without any treatment; LPS = cells treated with only 50 μg/mL LPS; LPS + ART = LPS (50 μg/mL) + artemisinin (20 μM).

To analyze the anti-inflammatory effects of artemisinin, pMECs were treated with 20 μM artemisinin for 12 h before being exposed to 50 μg/mL LPS for 24 h. As shown in Figure 4A,B, the mRNA expression abundance and content of inflammatory factors (IL-1β, TNF-α, and IL-6) induced by LPS were significantly downregulated (*p* < 0.05) by artemisinin; however, artemisinin supplementation did not affect (*p* > 0.10) LPS-induced IL-8 mRNA expression abundance. Thus, the anti-inflammatory effect of artemisinin was confirmed in LPS-induced pMECs.

### 3.5. Effects of Artemisinin on the NF-κB and MAPK Signaling Pathways in LPS-Induced PMECs

**Figure 5:**
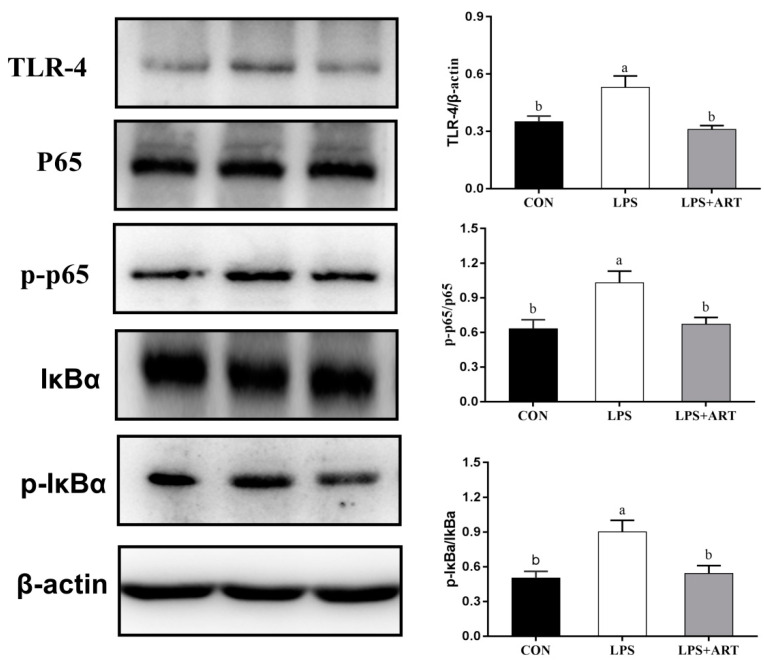
The effects of artemisinin on the expression of NF-κB signaling pathway-related proteins in LPS-induced pMECs. The data are the means ± SEM (*n* = 3). Different superscript letters (a,b) indicate that there are significant differences between the groups (*p* < 0.05). CON = control cells without any treatment; LPS = cells treated with only 50 μg/mL LPS; LPS + ART = LPS (50 μg/mL) + artemisinin (20 μM). Original western blot figures in Figure S1.

**Figure 6:**
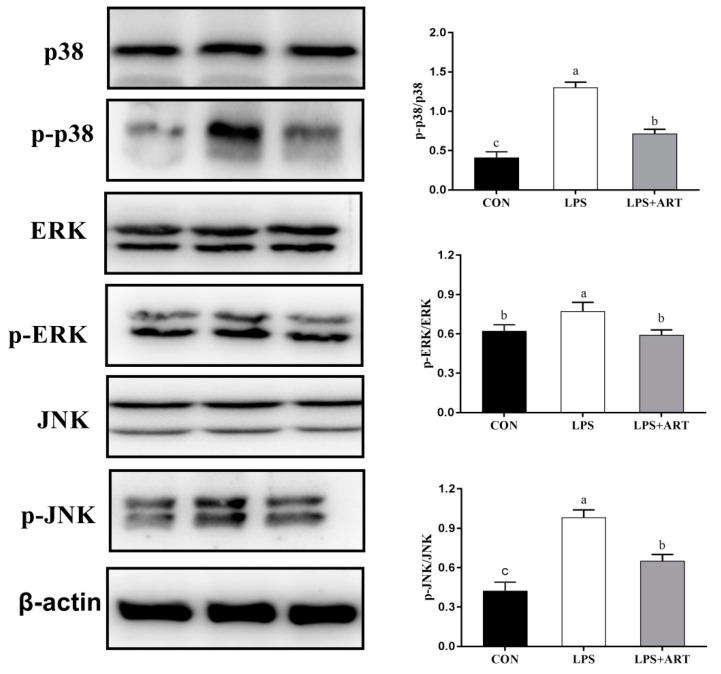
The effects of artemisinin on the expression of MAPK signaling pathway related proteins in LPS-induced pMECs. The data are the means ± SEM (*n* = 3). Different superscript letters (a,b,c) indicate that there are significant differences between the groups (*p* < 0.05). CON = control cells without any treatment; LPS = cells treated with only 50 μg/mL LPS; LPS + ART = LPS (50 μg/mL) + artemisinin (20 μM). Original western blot figures in Figure S2.

The nuclear factor κB (NF-κB) and mitogen-activated protein kinase (MAPK) signaling pathways are the most classic inflammatory signaling pathways. To determine the effect of artemisinin on the NF-κB and MAPK signaling pathways, we investigated the critical proteins of these signaling pathway by Western blot analysis. LPS activated cellular NF-κB and MAPK signaling pathways, causing inflammation. The phosphorylation levels of the NF-κB signaling pathway proteins p65 and IκBα were significantly increased (*p* < 0.05) (Figure 5), and the phosphorylation levels of the MAPK signaling pathway proteins p38, ERK and JNK were significantly increased (*p* < 0.05) (Figure 6) after the cells were treated with 50 μg/mL LPS for 24 h. When the cells were pretreated with 20 μM artemisinin for 12 h before being stimulated with 50 μg/mL LPS for 24 h, the phosphorylation levels of critical NF-κB and MAPK signaling pathway proteins were significantly decreased (*p* < 0.05; Figure 5 and Figure 6). These data showed that the activities of NF-κB and MAPK in LPS-induced pMECs were significantly inhibited by artemisinin.

## 4. Discussion

Escherichia coli (*E. coli*) is one of the main pathogens causing clinical mastitis in animals [32]. LPS is the main component of the outer membrane of *E. coli*, which is an effective initiator of inflammation and endotoxic shock. In this study, LPS was used to stimulate inflammatory response in porcine mammary epithelial cells in vitro to study the effects of artemisinin on sow mastitis. The results showed that, after pMECs were stimulated with 50 μg/mL LPS for 24 h, cell viability was significantly decreased, inflammatory factor mRNA and protein expression and apoptosis were significantly increased (Figure 1), suggesting that the cells exhibited an inflammatory response. This finding indicates that the pMECs inflammation model was successfully established.

Artemisinin has the potential anti-inflammatory effects [19], but the underlying mechanism of artemisinin in regulating inflammation has not been fully explained. Therefore, we studied the anti-inflammatory effect of artemisinin against LPS-induced inflammatory injury in pMECs and examined its mechanism. Previously, it has been confirmed that LPS stimulates mammary epithelial cells to produce a rapid inflammatory response, which is characterized by the release of a large number of pro-inflammatory cytokines (IL-1β, TNF-α, and IL-6) [33]. This response is good for attracting circulating immune effector cells (such as neutrophils) to fight infection, but excessive inflammation can damage cells and tissues [34]. Therefore, the expression of pro-inflammatory cytokines needs to be strictly regulated in the inflammatory response [35]. Studies have shown that artemisinin has a variety of biological functions, such as anti-inflammatory effects. Wang et al. [36] reported that artemisinin pretreatment potently inhibited IL-6 and TNF-αrelease induced by LPS in RAW264.7 cells, and artemisinin synergized with antibiotics to protect against LPS challenge by decreasing pro-inflammatory cytokine release. Yuan et al. [37] reported that proinflammatory factor levels (IL-1β, IL-6, TNF-α) and TLR-2 were significantly ameliorated by artemisinin treatment in LL37-induced rosacea-like mice, and artemisinin significantly decreased the LL37-induced expression of inflammatory biomarkers via the NF-κB signaling pathway in HaCaT cells. Li et al. [38] also reported that dihydroarteannuin can strongly reduce TNF-α levels in the culture supernatant of peritoneal macrophages and the serum of BXSB mice in vitro and in vivo, and the inhibitory effects on TNF-α production resulted from blockade of the NF-κB signaling pathway upstream of IκB degradation. Similarly, in this research, we examined the protective effect of artemisinin against LPS-induced inflammatory injury in pMECs and observed that the LPS-induced expression of IL-1β, TNF-α, and IL-6 was significantly down-regulated by artemisinin (Figure 4). It is worth noting that, in this experiment, we did not analyze the effects of artemisinin on inflammatory response without LPS challenge in pMECs, which is required to be addressed in the future.

**Figure 7:**
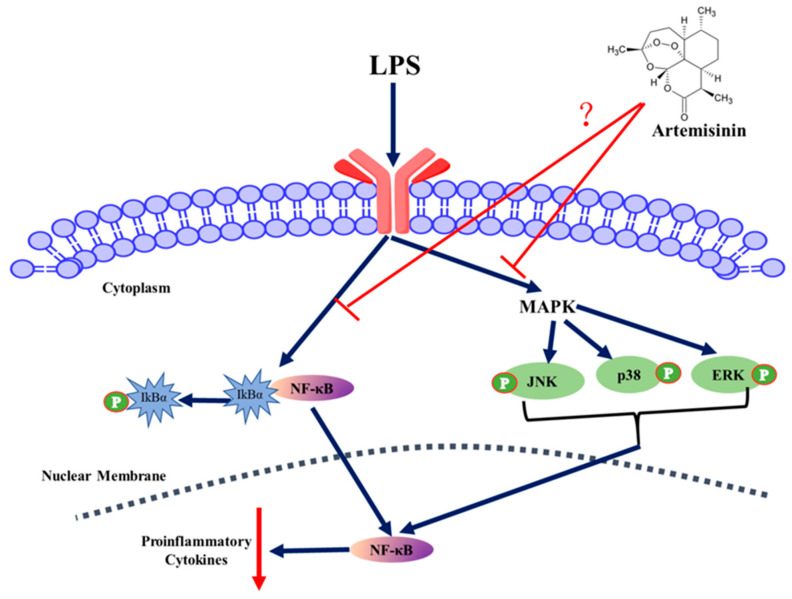
Possible mechanisms underlying the effects of artemisinin and LPS on pMECs. TLR4-LPS interaction leads to the rapid and coordinated activation of NF-κB and MAPK signaling pathways. Artemisinin reduces LPS-induced inflammatory damage to pMECs by inhibiting the MAPK and NF-κB signaling pathways.

In the present study, LPS-induced inflammatory damage to pMECs were weakened by artemisinin without cytotoxicity (Figure 3 and Figure 4). In addition, we identified artemisinin as an inhibitor of the NF-κB and MAPK signaling pathways. LPS leads to the rapid and coordinated activation of various intracellular signaling pathways, including NF-κB and MAPK signaling pathways [39]. NF-κB regulates the expression of chemokines, cytokines, anti-apoptotic factors and cell growth factors, and can also trans-activate various physiological and pathological processes, such as innate and adaptive immune responses, cell proliferation, cell death, inflammation and, in some cases, tumorigenesis [40,41]. MAPK contains at least three MAPK families (ERK, p38 and JNK), which could be activated and phosphorylated by LPS, thereby activating transcription factors during inflammation [42,43]. Thus, inhibiting NF-κB and MAPK activation can be used as a potential therapeutic strategy for treating inflammatory events [44,45]. Previous studies have shown that pretreatment of Hep3B cells with artemisinin significantly inhibited TNF-α-induced expression of the NF-κB reporter gene. Artemisinin can also inhibit TNF-α-induced degradation of IκBα and p65 nuclear translocation [46]. In addition, artemisinin could decrease the expression of the inflammatory proteins COX-2 and iNOS and effectively inhibit vascular smooth muscle cells inflammation induced by TNF-α through the NF-κB signaling pathway [47]. Zhu et al. [48] reported that artemisinin attenuates LPS-induced proinflammatory responses by inhibiting the NF-κB pathway in microglial cells. Aldieri et al. [43] also showed that artemisinin inhibited NF-κB activation in cytokine-stimulated human astrocytoma T67 cells. Mechanistically, artemisinin suppressed LPS-induced NF-κB activation by blocking IκBα degradation and p65 phosphorylation and inhibited MAPK activation by blocking ERK, JNK, and P38 phosphorylation (Figure 7). It would be interesting to investigate whether artemisinin could be used as a drug to treat LPS-induced inflammatory. In addition, future studies are needed to study the anti-inflammatory activity of artemisinin in an *in vivo* model of sow mastitis.

## 5. Conclusions

In summary, the pretreatment of pMECs with artemisinin showed enhanced anti-inflammatory activity against LPS-induced inflammation. Artemisinin reduces the inflammatory damage of pMECs partially through the inhibition of NF-κB and MAPK signaling pathways.

## Figures and Tables

**Table 1:** Primer sequences for the mRNA.

Genes	Accession	Sequence Primers (5′-3′)	Size (bp)
TNF-α	NM_214022.1	F-ATGGGCTGTACCTCATCTACTC	141
		R- GGCTCTTGATGGCAGAGAGG	
IL-1β	XM_021081828.1	F-CCGAAGAGGGACATGGAGAA	88
		R-AGTTGGGGTACAGGGCAGAC	
IL-6	NM_214399.1	F-TGGCTACTGCCTTCCCTACC	132
		R-CAGAGATTTTGCCGAGGATG	
IL-8	NM_213867.1	F-AGGACCAGAGCCAGGAAGAGAC	108
		R-CACAGAGAGCTGCAGAAAGCAG	
β-actin	XM_021086047.1	F-TGCGGGACATCAAGGAGAAG	176
		R-AGTTGAAGGTGGTCTCGTGG	

Abbreviations: TNF-α, tumor necrosis factor-α; IL-1β, interleukin-1β; IL-6, interleukin-6; IL-8, interleukin-8.

## Data Availability

All data used in this study can be obtained from the corresponding author according to the author’s reasonable request.

## Supplementary Materials

The following are available online at https://www.mdpi.com/article/10.3390/ani11061528/s1, Figure S1: Full original Western blot (WB) images for Figure 5, Figure S2: Full original Western blot (WB) images for Figure 6.
