# Supplementary material for: Artemisinin Protects Porcine Mammary Epithelial Cells against Lipopolysaccharide-Induced Inflammatory Injury by Regulating the NF-κB and MAPK Signaling Pathways
Source: Animals (Basel). 2021 May 24;11(6):1528. doi: 10.3390/ani11061528 (PMC8225056; doi:10.3390/ani11061528)
Supplement: Supplementary file 1 [file animals-11-01528-s001.zip › animals-1194462-supplementary.pdf]

Supplementary figure 1

(Figure 5)

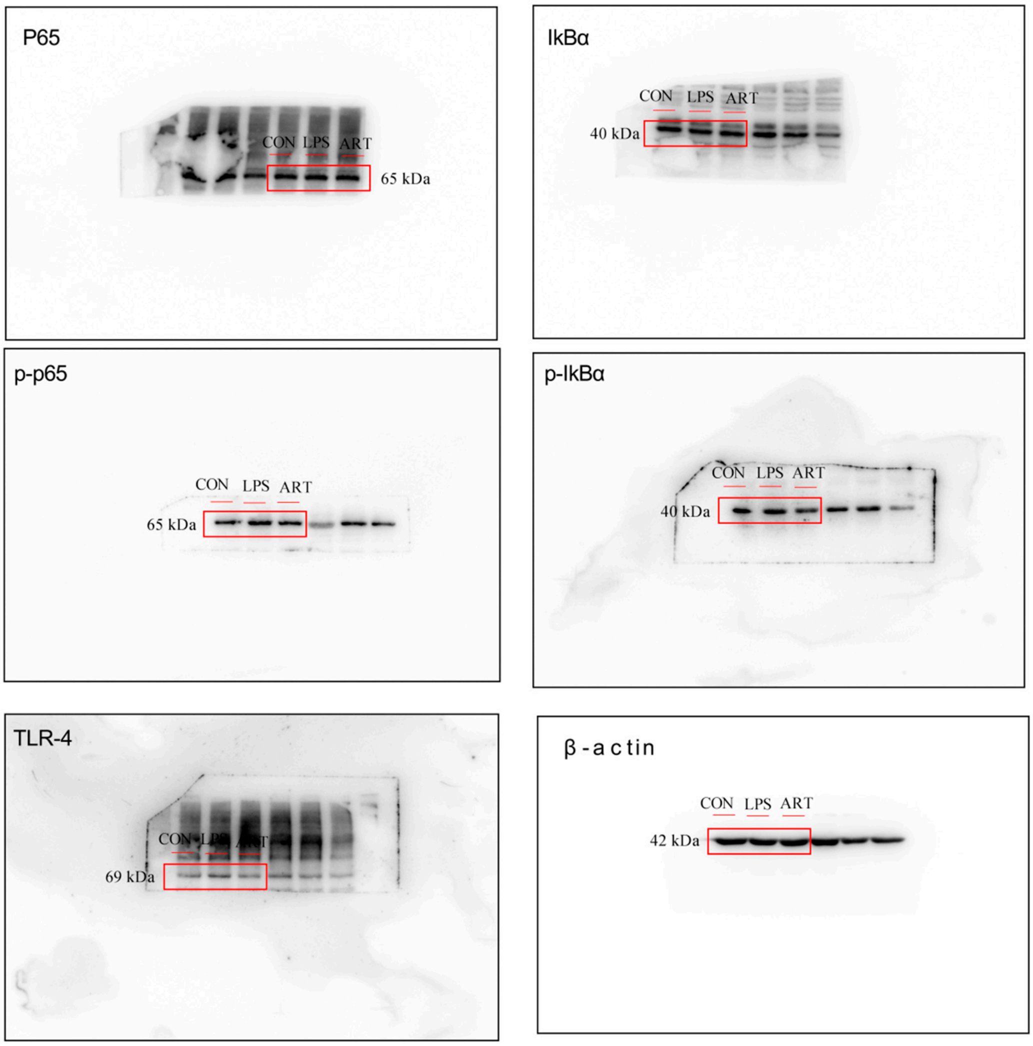

CON = control cells without any treatment; LPS = cells treated with only 50 μg/mL LPS; ART = LPS (50 μg/mL) + artemisinin (20 μM).

Figure S1. Full original Western blot (WB) images for Figure 5.

Supplementary figure 2

(Figure 6)

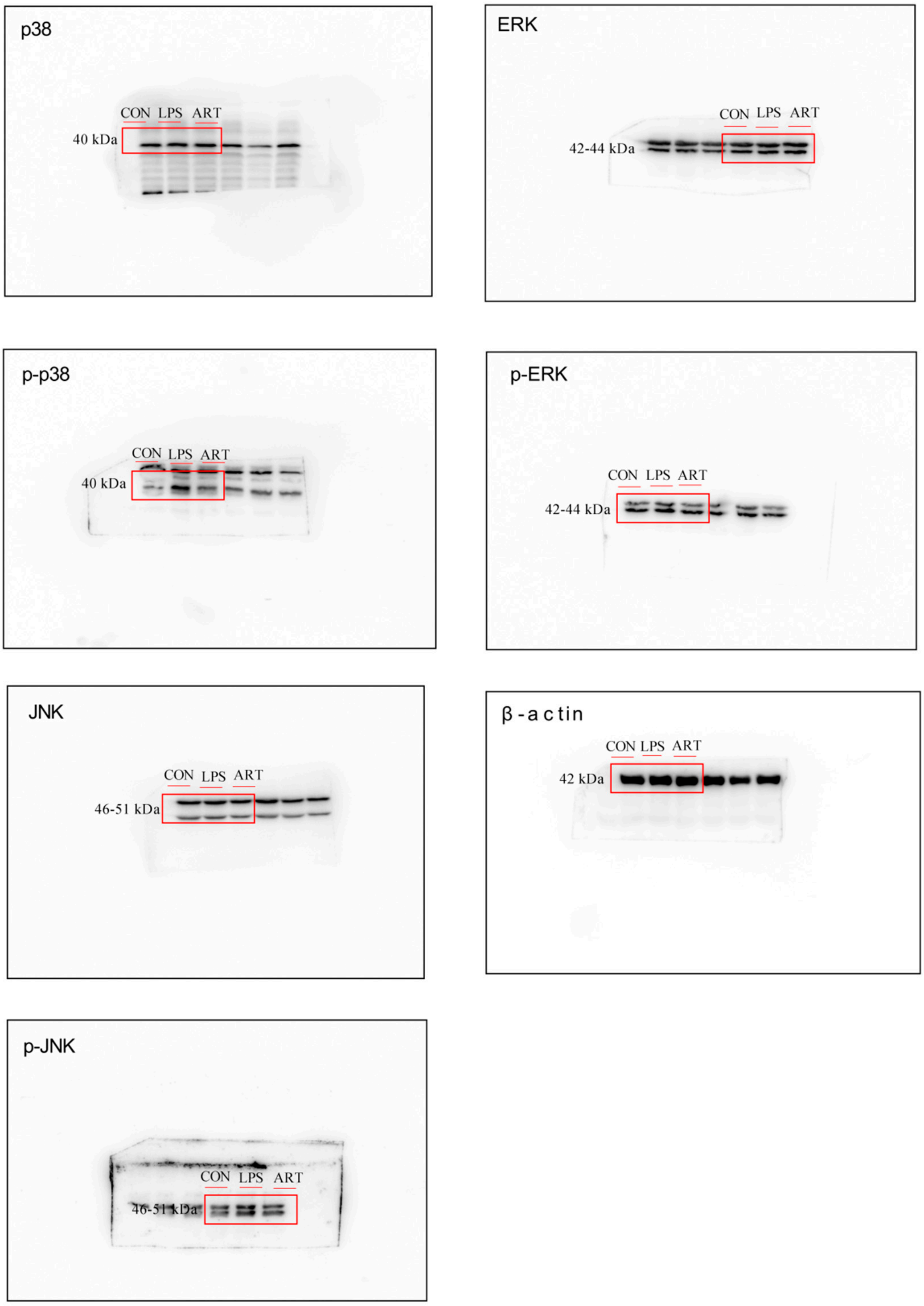

CON = control cells without any treatment; LPS = cells treated with only 50 μg/mL LPS; ART = LPS (50 μg/mL) + artemisinin (20 μM).

Figure S2. Full original Western blot (WB) images for Figure 6.
